# Expression of Chimeric HPV-HIV Protein L1P18 in *Pichia pastoris*; Purification and Characterization of the Virus-like Particles

**DOI:** 10.3390/pharmaceutics13111967

**Published:** 2021-11-20

**Authors:** Yoshiki Eto, Narcís Saubi, Pau Ferrer, Joan Joseph-Munné

**Affiliations:** 1 Department of Microbiology, Hospital Universitari Vall d’Hebron, 08035 Barcelona, Spain; yoshiki0522@gmail.com (Y.E.); narcis.saubi@vhir.org (N.S.); 2 Department of Chemical, Biological and Environmental Engineering, School of Engineering, Universitat Autònoma de Barcelona, 08193 Bellaterra, Spain; pau.ferrer@uab.cat; 3 Vall d’Hebron Research Institute, Hospital Universitari Vall d’Hebron, 08035 Barcelona, Spain; 4 AIDS Research Unit, Infectious Diseases Department, Hospital Clínic/IDIBAPS, School of Medicine, University of Barcelona, 08036 Barcelona, Spain

**Keywords:** HPV, HIV, virus-like particle, chimeric, vaccine, yeast, *Pichia pastoris*, purification

## Abstract

Currently, three human papillomavirus (HPV) vaccines are already licensed and all of them are based on virus-like particles (VLPs) of HPV L1 capsid protein but not worldwide accessible. While about 38.0 million people were living with HIV in 2019, only 68% of HIV-infected individuals were accessing antiretroviral therapy as of the end of June 2020 and there is no HIV vaccine yet. Therefore, safe, effective, and affordable vaccines against those two viruses are immediately needed. Both HPV and HIV are sexually transmitted infections and one of the main access routes is the mucosal genital tract. Thus, the development of a combined vaccine that would protect against HPV and HIV infections is a logical effort in the fight against these two major global pathogens. In this study, a recombinant *Pichia pastoris* producing chimeric HPV-HIV L1P18 protein intracellularly was constructed. After cell disruption, the supernatant was collected, and the VLPs were purified by a combination of ammonium sulfate precipitation, size exclusion chromatography, ultracentrifugation, and ultrafiltration. At the end of purification process, the chimeric VLPs were recovered with 96% purity and 9.23% overall yield, and the morphology of VLPs were confirmed by transmission electron microscopy. This work contributes towards the development of an alternative platform for production of a bivalent vaccine against HPV and HIV in *P. pastoris*.

## 1. Introduction

More than 100 types of human papilloma virus (HPV) are already known and the HPV genotypes 16 and 18 are considered to be responsible for about 70% of cervical cancer worldwide [1,2,3]. Currently, three HPV vaccines are licensed and all of them are based on virus-like particles (VLPs) of HPV L1 capsid protein [4,5,6]. Two of them, Gardasil and Gardasil 9 are produced by Saccharomyces cerevisiae (one type of yeast expression system) while the other one, Cervarix, is produced by baculovirus expression system. However, these three are still prohibitively expensive for the majority of women in developing countries and the cervical cancer caused by HPV is a lasting menace in those areas [7,8,9,10]. 

As for human immunodeficiency virus (HIV), in 2019, about 38.0 million people were living with HIV, 1.7 million individuals became newly infected with HIV, including 320,000 children and adolescents [11,12]. Approximately 70% of people living with HIV are in Sub-Saharan Africa. Effective treatment with antiretroviral therapy (ART) besides prevention of new infections among key populations have helped in the fight against the HIV/AIDS pandemic [13,14,15,16]. However, only 68% of HIV-infected individuals were accessing antiretroviral therapy as of the end of June 2020 [11]. Several types of HIV vaccine have been investigated for over 35 years, and a number of clinical trials have been held but the best obtained result so far is 31.2% vaccine efficacy in the clinical trial RV 144 [17]. The extraordinary diversity of HIV-1, the capacity of the virus to evade adaptive immune responses, the inability to induce broadly reactive antibody responses, the early establishment of latent viral reservoirs, and the lack of clear immune correlates of protection represent unprecedented challenges for vaccine development [18]. Both HPV and HIV are important public health issues in developing and industrialized countries and any reasonable prevention strategies are still not available, so a safe, effective and affordable vaccine is immediately needed [19,20,21,22]. 

*Pichia pastoris* (syn. *Komagataella phaffii*) is a well-studied methylotrophic yeast harboring an extensive toolbox for genetic engineering and is more easily scalable to industrial production than *S. cerevisiae* [23,24,25,26]. Specifically, it provides high levels of heterologous protein expression and rapid growth on relatively simple defined media to very high cell densities, so that it is cheap to use while it could give a better yield compared to other yeast expression systems thanks to the use of the strong Glyceraldehyde-3-phosphate dehydrogenase (GAP) promoter, which drives production of proteins constitutively. In addition, heterologous gene expression in *P. pastoris* by using DNA expression vectors require less manipulation than the baculovirus expression system, where one step of cell culture growth (sf9 or sf21 cells) and one step of infection and protein harvest are needed [27,28,29]. In this study, we aimed at developing a chimeric VLP-based HPV-HIV vaccine by using *P. pastoris*, a well-established yeast expression system. Here we describe the expression of chimeric HPV-HIV protein, HPV genotype 16 capsid protein L1 and HIV-1 CTL epitope P18-I10 (L1P18), in *P. pastoris*, and the purification and characterization of the virus-like particles. HPV genotype 16 L1 is the major structural protein of the virus capsid. Five of the L1 proteins form a pentamer and 72 of the pentamers self-assemble into a VLP [30,31,32]. HPV L1 VLPs have been already used as HPV vaccines and the vaccinations led to the stimulation of humoral immune responses [33,34,35]. The P18-I10 epitope was selected to be used as HIV-1 immunogen in HPV-VLP because, on top of being part of V3 loop of gp120, it can elicit antibodies responses and it is a murine H-2D^d^ -restricted CTL epitope (RGPGRAFVTI) useful to measure the specific HIV-1 T-cell mediated immunity in BALB/c mice [36]. On the other hand, we selected this epitope as a starting point and proof of concept experiment for the chimeric HPV/HIV VLP for HPV and HIV vaccine development platform.

A recombinant *P. pastoris* expressing the chimeric HPV-HIV L1P18 encoding gene was constructed using the expression vector pGAPZB for intracellular protein production. The recombinant yeast strains were genetically and phenotypically characterized. The L1P18 protein expression was confirmed by western blotting. After L1P18 proteins were produced intracellularly in the yeast, the cells were lysed and the VLPs were purified by a combination of ammonium sulfate precipitation, size exclusion chromatography, ultracentrifugation and ultrafiltration. After the purification, the morphology of VLPs were confirmed by transmission electron microscopy. Both HPV and HIV are sexually transmitted infections and one of the main access routes is the mucosal genital tract. Thus, the development of a combined vaccine that would protect against HPV and HIV infections is a logical effort in the fight against these two major global pathogens. In addition, *P. pastoris* could be a good alternative in terms of production costs and volume compared to superior eukaryotic expression systems for VLP production. Here we propose a novel approach for chimeric HPV-HIV VLP production and purification based on size exclusion chromatography that, in our hands, has proven to be more reliable and efficient compared to previously available approaches using affinity chromatography or ultracentrifugation, which were irreproducible in our study [37,38]. This work contributes towards the development of an alternative platform for production of a bivalent affordable vaccine against two viruses such as HPV and HIV, which is urgently needed in developing countries.

## 2. Materials and Methods

### 2.1. Bacterial and Yeast Strains, and Cultures

The *Escherichia coli* DH5α strain (Invitrogen - Thermo Fisher Scientific, Waltham, MA USA) was used as a host for plasmid DNA extraction and purification, cultured in low salt LB medium (1% tryptone, 0.5% NaCl, and 0.5% yeast extract plus 2% agar in plates), and supplemented with 25 μg/mL Zeocin (InvivoGen, San Diego, CA, USA) for the selection of transformants. The methylotrophic yeast *P. pastoris* X-33 strain (Invitrogen) was routinely cultured in YPD media (1% yeast extract, 2% peptone, and 2% dextrose, plus 2% agar in plates) and supplemented with 100 μg/mL Zeocin for the selection of transformants.

### 2.2. Design and Construction of Recombinant P. pastoris X-33 Strain Expressing Chimeric L1P18 Protein

The HIV-1 murine H-2D^d^-restricted CTL epitope P18I10 peptide (RGPGRAFVTI) from the V3 loop of gp120 was inserted into D-E loop of HPV genotype 16 capsid protein L1.The DNA coding sequence corresponding to the chimeric HPV-HIV protein L1P18 was codon optimized for *P. pastoris* usage and cloned into pGAPZB plasmid DNA as a EcoRI-XhoI fragment under the control of GAP promoter. The recombinant plasmid was desgined by Clone Manager (Sci Ed Software, Westminster, CO, USA). pGAPZB is an integrative expression vector that contains a Zeocin resistance gene as a selectable marker and the expression cassette harboring the GAP promoter, multicloning site and transcriptional terminator. The construction and DNA sequencing of the plasmid DNA was performed by Life technologies. pGAPZB and pGAPZB-L1P18 were linearized by SacI enzyme and transformed into *P. pastoris* X-33 strain by electroporation with an electric field pulse: 1.5 kV/cm, 50 μF and 200 Ω and linearized plasmid DNAs were integrated into Pichia genome by a single crossover event between the GAP promoter region on the pGAPZ vectors and the GAP promoter locus on *P. pastoris*’ genome. The wild type X-33 strain and the transformants were plated out onto YPD agar plates with or without 100 μg/mL Zeocin. In order to verify the correct integration of the expression cassette, the genomic DNA extracted from the isolated yeast colonies were amplified by Polymerase chain reaction (PCR) by using specific primers corresponding to L1P18 DNA coding sequence (Fw: 5′-ATGTCTCTTTGGCTGCCTAGTG-3′, Rev: 5′-TTACAGCTTACGTTTTTTGCGTTTAG-3′).

### 2.3. Production and Purification of L1P18 VLPs

The working stock of the yeast transformant was grown in 1.75 L YPD broth containing 100 μg/mL Zeocin in baffled flasks on a shaker at 150 rpm for 40 h at 30 °C (OD_600_ ≈ 20) and 45 g wet cell weight of the recombinant *P. pastoris* cells were harvested by centrifugation at 5000× *g* for 5 min and washed by PBS twice to remove YPD broth. Then the cells were resuspended in 100 mL of the volume of ice-cold break buffer (20 mM sodium phosphate, pH 7.2, 100 mM NaCl, 1.7 mM EDTA, 0.01% Tween 80), with protease inhibitor cocktail tablets (Roche, Basel, Switzerland). After the cell lysis by cell disruptor (Constant System, Daventry, UK) at 2 kbar twice, the sample was centrifuged at 10,000× *g* for 30 min. The cell lysate supernatant containing L1P18 protein was adjusted to 45% saturated ammonium sulfate and left stirred for 1 h at 4 °C, and the precipitated protein was pelleted at 12,000× *g* for 10 min at 4 °C. The pellet was resuspended in 10 mL of PBS + 0.01% Tween 80. To remove further contaminants, the ammonium sulfate precipitate was dialyzed against PBS + 0.01% Tween 80 and diluted 10 times in incubation buffer (10 mM sodium phosphate, pH 7.2, 150 mM NaCl + 0.01% Tween 80). This suspension was incubated at room temperature for 24 h, and the precipitated protein was removed by centrifugation at 12,000× *g* for 10 min, and the supernatant obtained. After filtered by 0.45 μm, the sample was further purified by size exclusion chromatography with HiTrap Capto Core 700 column (GE Health care, Chicago, IL, USA) on ÄKTA pure (GE Health care, Chicago, IL, USA). This chromatography system combines size exclusion and anion exchange. Large target molecules (>Mr 700,000) pass outside the bead, while smaller molecules enter the bead pores and are bound by anion exchange active sites. In other words, L1P18 monomers and pentamers entered the bead and were captured in the column, while the VLPs did not enter the beads and were eluted during sample application as flowthroughs. The flowthrough samples were pooled and ultracentrifuged at 28,000× *g* for 4 h with 25% and 70% sucrose cushions. The VLP containing 25% fraction and the interface fraction between 25% and 70% were further purified and concentrated about 280 times with a centrifugal filter device, Vivaspin 20 centrifugal concentrator 1000 kDa (Sartorius, Göttingen, Germany).

### 2.4. Immunodot Analysis

The fractions collected after size exclusion chromatography or ultracentrifugation were loaded on methanol-activated PVDF membrane as a dot and left until it gets soaked. The membrane was blocked with 5% skim milk in TBS-T, and probed with the anti-L1 mAb[CamVir 1] (Abcam, Cambridge, UK) and mAb to HIV-1 V3 loop (reference: EVA3012 was obtained from Centre for AIDS reagents, NIBSC, Potters Bar, UK) at dilution of 1:2000, and a secondary antibody directed against mouse IgG conjugated to Peroxidase. Bound antibodies were detected using an enhanced chemiluminescence detection kit (GE Health care, Chicago, IL, USA).

### 2.5. Sodium Dodecyl Sulphate–Polyacrylamide gel Electrophoresis and Western Blot Analysis

Yeast cell lysate supernatants or purified VLP samples were diluted in sodium dodecyl sulfate polyacrylamide gel electrophoresis (SDS-PAGE) sample buffer, boiled at 100 °C for 10 min, electrophoresed through 10% TGX Stain-Free SDS-PAGE gel (Bio-Rad, Hercules, CA, USA) and proteins were detected by Stain-Free technology (Bio-Rad, Hercules, CA, USA). Then gel was transferred to a PVDF membrane using a Bio-Rad semi-dry apparatus. The membrane was blocked with 5% skim milk in TBS-T and probed with the anti-L1 mAb [CamVir 1] (Abcam, Cambridge, UK) at dilution of 1:2000, and a secondary antibody directed against mouse IgG conjugated to Peroxidase. Bound antibodies were be detected using an enhanced chemiluminescence detection kit (GE Health care, Chicago, IL, USA).

### 2.6. Transmission Electron Microscopy of L1P18 VLPs

The sample concentrated by ultrafiltration was fixed with 4% paraformaldehyde for overnight and then was negatively stained with 2% Phosphotungstic acid. Grids were left to air-dry prior to examination with a transmission electron microscope operated at the Electron Microscopy Unit of the Scientific and Technology Centers of the University of Barcelona.

### 2.7. Heterologous Protein and Total Protein Quantification

The total amount of L1P18 protein in each purification step was quantified by densitometry of the dots after immunoblot analysis, using several concentrations of recombinant HPV16 L1 protein (ab119880, Abcam, Cambridge, UK) as positive controls. The total protein of the sample after ultrafiltration step was analyzed by using Pierce™ BCA Protein Assay Kit and the L1P18 protein obtained after all the purification steps in this study was quantified by comparing the total protein and the purity.

### 2.8. Ethics Statement

All the experiments were approved by the local Research Ethics Committee (Procedure 43.19, Hospital de la Vall d’Hebron, Universitat Autònoma de Barcelona).

## 3. Results

### 3.1. Construction of Recombinant P. pastoris X-33-L1P18I10 Strain

**Figure 1:**
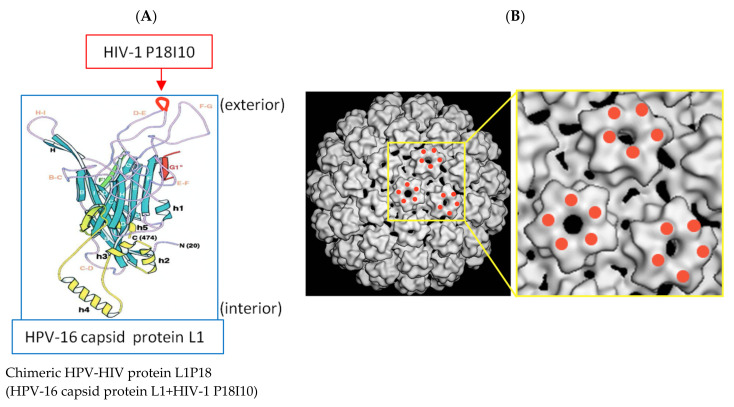
Immunogen design and construction of chimeric HPV-HIV VLP. (**A**) Chimeric HPV-HIV protein L1P18 model (Modified and adapted from [40]) (**B**) chimeric HPV-HIV virus-like particle (VLP) model (Modified and adapted from [41]).

**Figure 2:**
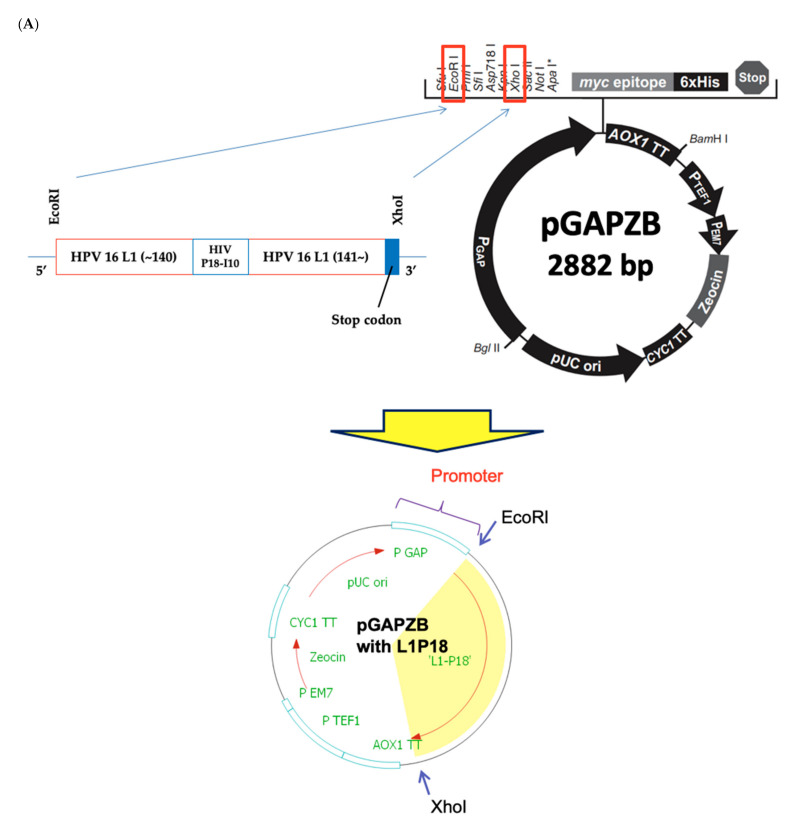

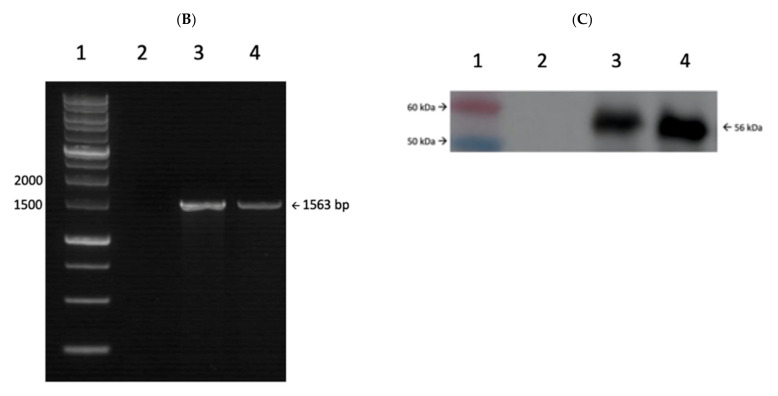
Construction of recombinant X-33-L1P18 yeast strain. (**A**) Cloning of L1P18 DNA coding sequence into yeast plasmid DNA pGAPZB. (**B**) PCR analysis of genomic DNA isolated from recombinant *P. pastoris* clones. Amplification of a 1563-bp DNA fragment containing L1P18 reveals positive clones. Lane 1, molecular weight markers; lane 2, Recombinant *P. pastoris* X-33 harboring the pGAPZB without heterologous DNA insert (negative control); lane 3, *P. pastoris* X-33 harboring the pGAPZB-L1P18 expressing the gene encoding the L1P18 protein; lane 4, pGAPZB-L1P18 plasmid (positive control) (**C**) Western blot analysis of recombinant *P. pastoris* cell lysate. Lane 1, molecular weight markers (50 kDa and 60 kDa); lane 2, Recombinant *P. pastoris* X-33 harboring the pGAPZB without heterologous DNA insert (negative control); lane 3, *P. pastoris* X-33 harboring the pGAPZB-L1P18 expressing the gene encoding the L1P18 protein; lane 4, abcam HPV16 L1 protein 100 ng (positive control, 56 kDa).

The HIV-1 P18I10 peptide was inserted into the loop D-E of the HPV-16 L1 protein because Sadeyen et al. [39] showed that Hepatitis B virus capsid (HBc) protein was more immunogenic when it was inserted in the D-E loop of HPV-16 L1 compared to other loops (Figure 1A). The D-E loop is exposed to the exterior when 360 L1 proteins form a VLP so that the inserted HIV-1 P18I10 proteins are supposed to be situated on the surface of the chimeric HPV-HIV VLP (Figure 1B). Using this design, the P18 immunogen is supposed to be presented 360 times in each VLP particle in a highly exposed location. The DNA fragment containing the L1P18 coding sequence was inserted into the integrative plasmid pGAPZB (Figure 2A), which constitutively drives production of proteins intracellularly, as a EcoRI-XhoI fragment under the control of glyceraldehyde-3-phosphate dehydrogenase (GAP) promoter. The constructed recombinant plasmid DNA was confirmed by Sanger sequencing (data not shown). The recombinant plasmid DNA was first transformed into *E. coli* by heat-shock for the amplification and purification of the plasmid DNA, and then transformed into the *P. pastoris* X-33 by electroporation. The integration of the expression vector into the genome of the transformants was verified by PCR analysis using a pair of primers flanking the L1P18 gene (Figure 2B). A band of 1563 bp corresponding to L1P18 DNA coding sequence was detected. Finally, L1P18 protein expression was further confirmed by western blot analysis and a band of 56 kDa corresponding to L1P18 protein was observed (Figure 2C).

### 3.2. Genetic and Phenotypic Characterization of Recombinant X-33-L1P18I10 Yeast Strain

**Figure 3:**
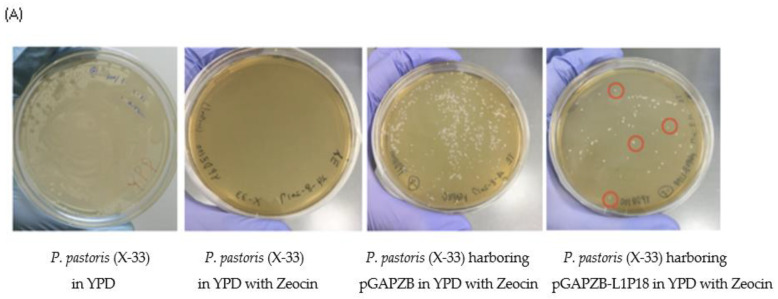

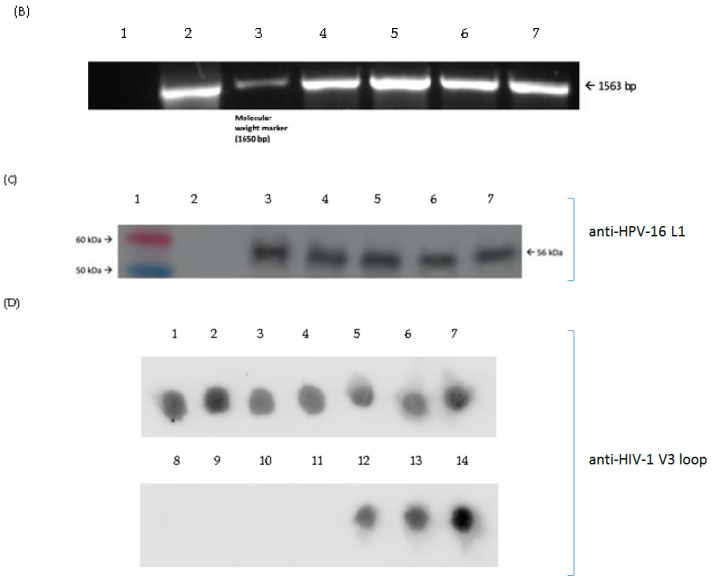
Genetic and phenotypic characterization of recombinant *P. pastoris*. (**A**) antibiotic selection of recombinant strains. 4 colonies of *P. pastoris* X-33 harboring pGAPZB-L1P18 (red circled) were used for yeast colony PCR. (**B**) Yeast colony PCR. Lane 1. X-33 harboring the pGAPZB without DNA insert (negative control); lane 2, plasmid pGAPZB-L1P18 (positive control); lane 3. Molecular weight marker (Invitrogen 1kb plus DNA ladder, 1650 bp); lanes 4–7: Recombinant yeast strains containing the pGAPZB-L1P18 carrying the DNA coding sequence L1P18 (1563 bp). (**C**) Western blot analysis of L1 protein expression in cell lysates of four recombinant clones of *P. pastoris* X-33 harboring pGAPZB-L1P18, probed with the anti-L1 mAb [CamVir 1] (Abcam, Cambridge, UK). Lane 1. Molecular weight markers (50 kDa and 60 kDa); lane 2, Recombinant *P. pastoris* X33 harboring the pGAPZB without heterologous DNA insert (negative control); lane 3, abcam HPV16 L1 protein 75 ng (positive control, 56 kDa); lanes 4–7, 4 recombinant *P. pastoris* X-33 colonies harboring the pGAPZB-L1P18 expressing the L1P18 protein. (**D**) Dot blot analysis of P18 peptide expression in cell lysates of two recombinant clones of *P. pastoris* X-33 harboring pGAPZB-L1P18, probed with mAb to HIV-1 V3 loop (reference: EVA3012, Center for AIDS reagents, NIBSC). Dot 1, 2: protein extracted from recombinant BCG:HIVA 222 strain expressing HIVA immunogen that contains P18 peptide (4, and 8 µL) (positive control); dot 3 and 4: protein extracted from recombinant BCG:HIVA 223 strain (4 and 8 µL) (positive control); dot 5, 6 and 7: cell lysate of recombinant *P. pastoris* clone#3 (4, 8 and 12 µL); dot 8, PBS (negative control); dot 9, Abcam recombinant HPV16 L1 protein (ab119880) (50 ng) (negative control); dot 10, Abcam recombinant HPV16 L1 protein (ab119880) (20 ng) (negative control); dot 11, Abcam recombinant HPV16 L1 protein (ab119880) (10 ng) (negative control); dot 12, 13 and 14: cell lysate of recombinant *P. pastoris* clone #4 (4, 8 and 12 µL).

Once the recombinant *P. pastoris* X-33-L1P18I10 strain was constructed, the master Seed and the working stock was generated. Taking into account clone heterogeneity, a phenomenon commonly found when transforming *P. pastoris* (e.g., due to seldom integration of multiple copies of the expression vector, ectopic integration events or post transformation recombination events), four independent yeast clones harboring pGAPZB-L1P18 were selected by using Zeocin antibiotic selection system (Figure 3A). Since pGAPZB is an integrative plasmid DNA, the yeast genomic DNA was extracted from the cell culture, and the integration of the expression vector containing the L1P18 DNA coding sequence in the recombinant yeast colonies was confirmed by PCR analysis and using purified genomic DNA from the transformants as template (Figure 3B). A band of 1563 bp corresponding to L1P18 DNA coding sequence was detected. These recombinant yeast colonies were further analyzed to test L1P18 production levels. To this end, clones were picked up from the agar plate. The L1P18 protein production was confirmed from lysed yeast cells by western blot analysis (Figure 3C). In addition, the expression and reactivity of HIV-1 P18 peptide was detected by immunodot analysis (Figure 3D). L1P18 production level among four clones were the same so that they were stored as working stocks for further use.

### 3.3. Production and Purification of Chimeric L1P18 VLPs

**Figure 4:**
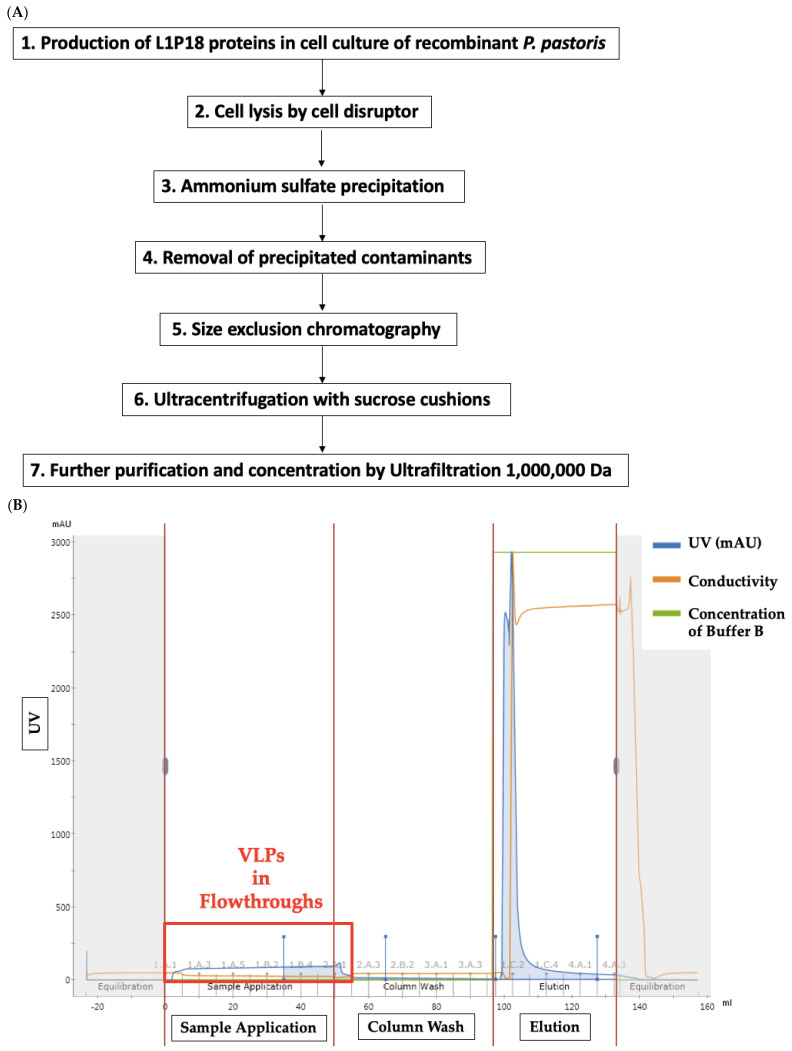

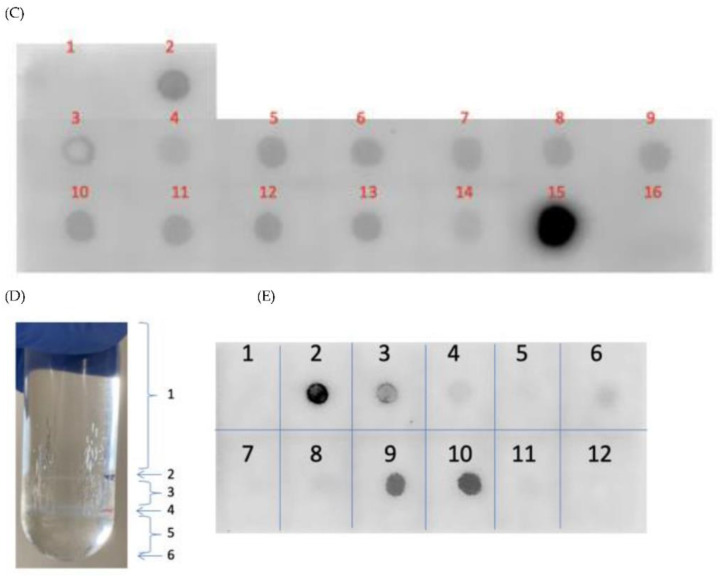
Production and purification of chimeric L1P18 VLPs. (**A**) Flowchart of the L1P18 VLPs production and purification process designed and performed successfully in this study. (**B**) Size exclusion chromatogram after removal of precipitated contaminants. The flowthrough fractions containing L1P18 VLPs were collected. (**C**) Immunodot analysis of flow through and elution fractions after size exclusion chromatography. Dot 1, PBS (negative control); dot 2, 2.5 ng L1 protein (positive control); dot 3. Pre-size exclusion chromatography sample; dots 4–14 flowthrough fractions; dots 15 and 16. Elution fractions. The extremely high intensity of dot 15 is due to the sample concentration at elution. L1P18 monomers and pentamers in 50 mL sample were eluted in 5 mL sample. (**D**) Fractions after ultracentrifugation. Fraction 1, 0% sucrose; fraction 2, interface between 0% and 25% sucrose; fraction 3, 25% sucrose; fraction 4, interface between 0% and 25% sucrose; fraction 5, 70% sucrose; fraction 6, pellet resuspended 0.1 mL breaking buffer. (**E**) Immunodot analysis of post-ultracentrifugation fractions. dot 1, PBS; dot 2, abcam HPV-16 L1 20 ng (+control); dot 3, abcam HPV-16 L1 10 ng (+control); dot 4, abcam HPV-16 L1 5 ng (+control); dot 5, abcam HPV-16 L1 2.5 ng (+control); dot 6. Pre-ultracentrifugation sample; dot 7, 0% sucrose; dot 8, Interface between 0% and 25%; dot 9, 25% sucrose; dot 10, Interface between 25% and 70%; dot 11, 70% sucrose; dot 12, pellet resuspended with 0.1 mL breaking buffer.

The yeast cells were Approximately, 1 mg of L1P18 protein was produced in 45 g wet cell weight of *P. pastoris* (OD_600_ ≈ 20) from the 1.75 L shake flask culture (data not shown). After systematic search for an effective purification method, the procedure shown in Figure 4A was obtained. In the size exclusion chromatography, chimeric L1P18 VLPs from the monomers and pentamers were separated in flow through fractions (Figure 4B). The total volume sample (200 mL) was processed in 50-mL runs. The dot blot analysis confirmed that the flowthrough fractions contained the L1P18 proteins likely to be assembled as VLPs and the elution fractions contained L1P18 as monomers and pentamers (Figure 4C). The flowthrough fractions were pooled and ultracentrifuged for further purification (Figure 4D). Dot blot analysis demonstrated that L1P18 VLPs were trapped in the 25% sucrose fraction and the interface fraction between 25% and 70% sucrose cushions (Figure 4E). The 25% sucrose fraction and the interface fraction between 25% and 70% sucrose cushions were filtered by 0.45 µm and then further purified and concentrated about 280 times (28 mL → 0.1 mL) by using 1000 kDa cut-off ultrafiltration device. This cut-off line was selected because the molecular mass of the L1P18 monomer, pentamer and VLP is 56 kDa, 280 kDa, and 20,160 kDa, respectively, and therefore, 1000 kDa ultrafiltration device holds the VLPs while the monomers and pentamers pass through. The total amount of L1P18 protein in each purification step was quantified by densitometry of dots against L1 antibody in dot blot analysis (data not shown) comparing with different amounts of abcam HPV16 L1 protein as positive controls (Table 1). Recovery yields were calculated, based on the amount of total L1P18 protein in cell lysate supernatants, considering it as 100%.

### 3.4. Characterization of Purified L1P18 VLPs

**Figure 5:**
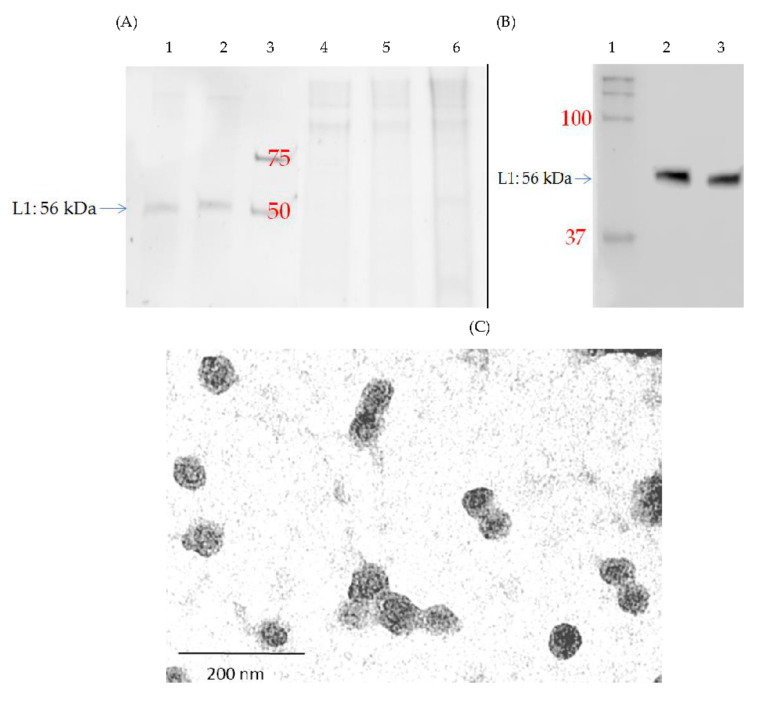
Characterization of purified L1P18 VLPs. (**A**) Coomassie staining of purified L1P18 VLPs. Lane 1, abcam HPV-16 L1 1 μg (+control); lane 2, purified L1P18; lane 3, BIO-RAD Precision Plus Protein™ WesternC™ Standards 1 μL; lane 4; 25% sucrose fraction after ultracentrifugation; lane 5, Interface fraction between 25% and 70% after ultracentrifugation; lane 6, mixture of 25% sucrose fraction and the interface fraction between 25% and 70% sucrose fractions after ultracentrifugation. (**B**) Western blot analysis of purified L1P18 VLPs. lane 1, BIO-RAD Precision Plus Protein™ WesternC™ Standards 5 μL; lane 2, 10x diluted purified L1P18 VLPs; lane 3, abcam HPV-16 L1 100 ng (+control). (**C**) Transmission electron microscopy of purified HPV-HIV L1P18 VLPs (magnification 54,000× and bar 200 nm).

The Coomassie staining of SDS-PAGE analyses showed that the contaminants over 75 kDa seen in the 25% sucrose fraction and the interface fraction between 25% and 70% sucrose cushions (Figure 5A, lanes 4–6) were removed by ultrafiltration and L1P18 was also concentrated and purified (Figure 5A, lane 2). The estimated purity of L1P18 (Figure 5A, lane 2) is 96%.

The purified and concentrated sample after ultrafiltration was 10 times diluted with PBS. Western blot analysis confirmed the heterologous L1P18 protein expression in the sample after ultrafiltration (Figure 5B, lane 2) which is similar in size (56 kDa) with the positive control abcam HPV16-L1 (Figure 5B, lane 3). The sample after ultrafiltration was analyzed by BCA protein assay and the concentration of total protein was 78.1 µg/mL. Therefore, the total amount of protein is 7.81 µg. As the purity of L1P18 was 96% according to Coomassie staining result, 7.50 µg of HPV-HIV L1P18 VLPs was purified from 1.75 L YPD culture. Considering that the amount of L1P18 protein in the cell lysate supernatant was 81.3 µg (data not shown), the recovery yield was 9.23%. The morphology of the chimeric L1P18 VLPs was observed under transmission electron microscope at magnification of 54,000×. Identical particles of diameter of ~50 nm were observed (Figure 5C).

## 4. Discussion

The development of an affordable, safe, and effective preventive vaccine against HPV and HIV is still an urgent need. In this study, (i) the recombinant *P. pastoris* X-33 strain expressing chimeric L1P18 protein were constructed and genetically and phenotypically characterized; (ii) the heterologous L1P18 protein production and reactivity against L1 protein and HIV-1 P18 peptide was confirmed; (iii) the chimeric HPV-HIV VLPs were successfully generated by using the yeast expression system; (iv) the chimeric HPV-HIV L1P18 VLPs were purified by ammonium sulfate precipitation, size exclusion chromatography, ultracentrifugation and ultrafiltration; (v) the recovery yield and purity degree were assessed; vi) purified chimeric VLPs were characterized and the morphology and size of the chimeric VLPs was confirmed by TEM. The recombinant *P. pastoris* X-33 strain expressing chimeric L1P18 protein was developed in Good Laboratory Practices (GLP)-compatible conditions, preserved according to the seed-lot system, and was genetically and phenotypically characterized. Overall, we have demonstrated that chimeric HPV-HIV VLPs produced by *P. pastoris* were successfully purified. Thus, this strategy might be worthy to pursue and for joining the global efforts to develop novel chimeric VLP vaccines for controlling HPV and HIV infection.

All three commercialized HPV VLP vaccines, Cervarix, Gadasil, and Gardasil 9, are based on baculovirus/insect cell expression system or yeast expression system. Regarding the expression systems for the chimeric VLP production, we chose to use yeast expression system because of its ease of handling and less cost compared to baculovirus/insect cells. Recently, human embryonic kidney cells (HEK) 293 cells and *E. coli* are also used for HPV VLP production, and they showed that they have great potential to be a better production system for the future HPV VLP vaccines and HPV-HIV chimeric VLP vaccines [42].

As recently discussed by Eto et al. in a review article [43], despite the fact there is no VLP-based HIV vaccine in the market, several prototypes of HIV VLPs have already been constructed. According to these studies, VLP-based HIV vaccine can elicit higher level of HIV-1-specfic neutralizing antibodies and CTL immune responses, particularly when administered mucosally [44].

In this study, although about 1 mg of the recombinant L1P18 protein was produced in 45 g wet cell weight of *P. pastoris* (OD_600_ ≈ 20) (data not shown), only 81.3 µg of L1P18 protein was recovered in the cell lysate supernatant after centrifugation. Setting this total amount of L1P18 protein in the lysate supernatant as a starting point, by our purification method, the purity of the VLPs reached 96% while the recovery yield was 9.23%. Comparing with two other similar purification studies [45,46], in which they purified HPV 16 L1 protein from *Saccharomyces cerevisiae* and the purities were 98.5% and 100% and the final recovery yields were 30.6% and 63% respectively, our recovery yield is lower while the purity is very similar. This could be due to the insertion of HIV epitope and the difference of yeast species. The critical point of the process for this chimeric VLPs production was to establish a purification method, by which the purity of the VLPs would reach over 95%. For this matter, a number of purification steps were systematically tested. In the process of this study, we encountered the following limitations, which would consider to improve for the future studies: (i) it was difficult to recover L1P18 protein right after cell lysate; (ii) The attempt to secrete L1P18 protein from *P. pastoris* was not successful; (iii) Ammonium sulfate precipitation had a lower recovery yield with our strain compared to previously published studies; (iv) it was challenging to reproduce the similar results to the published articles [45,46,47] with our recombinant protein by several chromatography purification methods. A major fraction (close to 90%) of the intracellularly produced protein may remain in the insoluble fraction after cell disruption, and several attempts showed that it was difficult to recover. In order to explore the capability of *P. pastoris* to secrete L1P18 protein/VLPs, we also constructed a recombinant *P. pastoris* harboring the integrative plasmid vector pPICZαA, which contains a secretion signal coding sequence, to express the L1P18 encoding gene regulated under the strong methanol-inducible promoter. As a reference, Law KH et al. [48] reported that 300 mg of *Streptomyces clavuligerus* β-lactamase inhibitory protein was secreted from 1 L of *P. pastoris*. However, in our case, a major challenge for efficient production of VLP in yeast was inability to efficiently secrete the heterologous protein. While a small amount of secreted L1P18 proteins were detected in liquid culture media, the majority of L1P18 protein were trapped inside the cells based on dot blot analysis (not shown). Moreover, no assembled VLPs were observed in the cytoplasm or other organelles (endoplasmatic reticulum, golgi, secretory vesicles, periplasm/cell wall), neither in the intracellular nor in the extracellular production strain. We suppose that L1P18 protein is presumably too big in size for efficient secretion, in other words, beyond *P. pastoris* secretion capacity. Based on the existing knowledge on protein secretion in yeast [49], the L1P18 protein is likely to block some step along the protein secretory pathway (either translocation into the ER, and/or traffic from ER to Golgi) and most of the produced L1P18 proteins with the secretion signal ends up being accumulated inside the yeast cells. Therefore, while Coimbra et al. [50] confirmed secreted L1 protein by immunodot, the secreted amount might be limited due to this blockage. An attempt to circumvent a potential bottleneck in the ER translocation step, different secretory signals were tested [51]. Nonetheless, these exploratory experiments have set the pathway for future cell engineering efforts towards VLP production in *P. pastoris*, which are being pursued by our collaborator group.

In terms of the limitations of our yeast cell factory of choice, regarding the potential glycosylation of the heterologous antigen, we should mention, that indeed *P. pastoris* does not produce proteins with humanised glycosylation profiles unless glycoengineered strains are used. Nonetheless, our expression strategy does not target the P18-I10 HIV-1 to the secretory pathway and, therefore, it cannot be glycosylated.

Regarding the contextualisation of our results, these are indeed a baseline study for the establishment of a yeast-based HPV-VLP platform for antigen presentation. The purification process originally reported for HPV VLP production from *S. cerevisiae* had an overall yield of 10% [52]. Although this yield could be sufficient for subsequent scale-up and commercialization of the product, it has been probably optimised over the years to reduce manufacturing costs.

In our system, some bottlenecks are still identified, namely an important fraction of product is retained in the insoluble fraction of the cell lysate. This could be reduced by optimising both the expression system (e.g., choice of promoter strength, co-expression of folding helpers) and the cultivation protocol. Also, first attempts of high-cell density cultivations at 5-L scale in our lab have proven that volumetric productivities (mg of product per litre of culture and time) can be largely improved (unpublished data). Introduction of primary recovery steps such as two-aqueous separation systems may also be applied to facilitate subsequent chromatographic steps [53], as well as circumventing the use of ultracentrifugation step for the final polishing step. Overall, we envisage that the combination of these strategies should allow for an efficient and competitive yeast platform for VLP production. Lastly, a great challenge still remains, i.e., the engineering of *P. pastoris* secretory pathway to allow efficient secretion of VLPs. This would further reduce the manufacturing costs of this type of platforms.

In this project, as a first VLP purification step, we chose to use ammonium sulfate precipitation instead of ultracentrifugation in order to have higher recovery of L1P18 protein, and to be able to handle large volumes, thinking of possible scaling up. Park et al. [46] showed that the VLP purification by ammonium sulfate precipitation showed maximally 15 times greater yields compared to the one by ultracentrifugation on sucrose cushion. Moreover, we tried several published purification methods [45,46,47], and it was difficult to reproduce the expected results by those methods and achieve a good purification yield and a high purity. For instance, Kim et al. [45] demonstrated that the recovery yield of HPV L1 protein, produced in *Saccharomyces cerevisiae*, by “ammonium sulfate precipitation and removal of precipitated protein” after cell lysate supernatant was 88%. However, our recovery yield of L1P18 protein produced in *P. pastoris* reached only 56.8%. Also, heparin chromatography, cation exchange chromatography, and anion exchange chromatography and combinations of them besides size exclusion chromatography were tested in order to optimize purification. While they seem ideal for purification of larger number of samples compared to ultracentrifugation, it was not successful to reproduce the similar results to the published articles [45,46,47] with our recombinant protein.

Therefore, we have systematically searched alternative methods for each of the stages, from early recovery to purification and final polishing. We have introduced changes in the sequence of purification steps and adding alternative steps, tested changes one by one in a systematic way in terms of introduction/substitution of conditioning stages (e.g., dialysis, UF), operating conditions (e.g., flow rates, elution gradients) and introduction/substitution of chromatographic steps, and characterized each alternative process in terms of purity, quantity, and yields. Finally, we have achieved a process that meets at least two of the objectives: (1) minimum degree of purity in order to reconstitute proper VLPs and (2) reproducible and robust process. However, as a future work, the methods still can be improved. For example, one bottleneck is the low protein recovery after yeast cell lysis, after using the intracellular protein expression vector, where the majority of L1P18 proteins stayed in insoluble fractions. We tried to solve this by using extracellular secreted protein by means of a secreting signal, but it was not successful, so we decided to pursue the cytosolic protein production. Overall, while our purification method reached 96% purity, the recovery yield was only 9.23%. Therefore, new purification methods must be investigated in order to improve the recovery. For instance, we cannot ignore the loss of a significant amount (approximately 65%) of L1P18 monomers and pentamers during the size exclusion chromatography step. Thus, a method with which these monomers and pentamers could be also purified and collected must be sought. Considering the fact that ultracentrifugation cannot be carried out on an industrial scale, an alternative purification process needs to be implemented such as heparin, cation exchange, and anion exchange chromatographies.

Our study is part of a cross comparison of VLP production in different platforms, including mammalian and insect cells. Next generation of chimeric HPV/HIV VLPs will be constructed by our group. In our case by using *P. pastoris* expression system and taking the L1 protein from HPV as a backbone to expose other HIV-1 B and T-cell epitopes to induce higher and robust specific-HIV-1 immunity. In fact, we have built-up chimeric VLPs L1-T20 by using the eukaryotic expression system and induced specific-HIV-1 and HPV antibodies in BALB/c mice (data not shown). T-20 is a HIV-1 peptide that contains a highly conserved linear epitope 2F5. The anti 2F5 antibodies have been reported to have broadly neutralizing activities. Therefore, we believe it is plausible that yeast-derived VLPs (assembled in vitro) will likely be able to trigger an immune response.

In conclusion, we successfully constructed, purified and characterized a VLP-based chimeric HPV-HIV vaccine candidate generated by using the *Pichia pastoris* expression system. Notably, we have demonstrated reactivity of chimeric L1P18 protein against anti-L1 and anti-HIV-1 V3 loop. Previous studies have shown that the insertion of the HBc antigen in D-E loop of HPV L1 protein can elicit specific immune responses [39]. It is plausible that our chimeric immunogen design might also induce specific HPV and HIV-1 immune responses. Further studies to evaluate the immunogenicity in small animal models and other biochemical studies such as peptide mapping, dynamic light scattering, super resolution microscopy, and temperature stability should be pursued. To the best of our knowledge, this is the first time that a systematic optimization of purification methods for chimeric HPV-HIV VLPs produced in *Pichia pastoris* was described. This approach might be applied to construct chimeric VLP vaccine candidates for other major pathogens.

## Figures and Tables

**Table 1:** Purification of recombinant L1P18 protein.

Step	Total Protein (µg) ^a^	Total L1P18 (µg)	Recovery Yield (%)	Purity (%) ^c^
Cell lysate supernatants	-	81.3 ^b^	100	-
Ammonium sulfate precipitation/ removal of precipitated contaminants	-	46.2 ^b^	56.8	-
Size exclusion chromatography	-	12.4 ^b^	15.3	-
Ultracentrifugation	-	9.57 ^b^	11.8	-
Ultrafiltration	7.81	7.50 ^d^	9.23	96%

^a^: determined by BCA protein assay. ^b^: determined by densitometry of dot blot analysis. ^c^: determined by densitometry of Coomassie-stained SDS-PAGE gel. ^d^: determined by total protein and purity.

## Data Availability

Not applicable.
